# Differential temporal release and lipoprotein loading in *B. thetaiotaomicron* bacterial extracellular vesicles

**DOI:** 10.1002/jev2.12406

**Published:** 2024-01-19

**Authors:** Rokas Juodeikis, Carlo Martins, Gerhard Saalbach, Jake Richardson, Todor Koev, Dave J. Baker, Marianne Defernez, Martin Warren, Simon R. Carding

**Affiliations:** ^1^ Food, Microbiome, and Health Research Programme Quadram Institute Bioscience Norwich UK; ^2^ Proteomics Facility John Innes Centre Norwich UK; ^3^ School of Pharmacy University of East Anglia Norwich UK; ^4^ School of Biosciences University of Kent Canterbury UK; ^5^ School of Biological Sciences University of East Anglia Norwich UK; ^6^ Norwich Medical School University of East Anglia Norwich UK

**Keywords:** bacterial extracellular vesicles, Bacteroides, BEV, OMV, outer membrane vesicles, proteomics

## Abstract

Bacterial extracellular vesicles (BEVs) contribute to stress responses, quorum sensing, biofilm formation and interspecies and interkingdom communication. However, the factors that regulate their release and heterogeneity are not well understood. We set out to investigate these factors in the common gut commensal *Bacteroides thetaiotaomicron* by studying BEV release throughout their growth cycle. Utilising a range of methods, we demonstrate that vesicles released at different stages of growth have significantly different composition, with early vesicles enriched in specifically released outer membrane vesicles (OMVs) containing a larger proportion of lipoproteins, while late phase BEVs primarily contain lytic vesicles with enrichment of cytoplasmic proteins. Furthermore, we demonstrate that lipoproteins containing a negatively charged signal peptide are preferentially incorporated in OMVs. We use this observation to predict all *Bacteroides thetaiotaomicron* OMV enriched lipoproteins and analyse their function. Overall, our findings highlight the need to understand media composition and BEV release dynamics prior to functional characterisation and define the theoretical functional capacity of *Bacteroides thetaiotaomicron* OMVs.

## 1 INTRODUCTION

The obligate anaerobic, Gram‐negative *Bacteroides* are an important genus of human gut commensal bacteria, which have a close relationship with their mammalian hosts (Ley et al., 2008; Wexler, 2007; Wexler & Goodman, 2017). *Bacteroides thetaiotaomicron* is a widely used model organism to study *Bacteroides*, particularly in understanding their extensive capacity to break down complex carbohydrates (Briliūtė et al., 2019; Lapébie et al., 2019). The bacterium releases a large number of bacterial extracellular vesicles (BEVs) which contain a specific proteome and interact with their environment (Durant et al., 2020; Jones et al., 2020; Juodeikis et al., 2022; Valguarnera et al., 2018).

BEVs are membranous vesicles released by many different types of bacteria. Depending on how they are formed these vesicles can be divided into two types: B‐type (non‐lytic) and E‐type (lytic) (Toyofuku et al., 2019; Toyofuku et al., 2023). As Gram‐negative bacteria, such as *B. thetaiotaomicron*, contain two membranes, the vesicles they produce can be further divided based on the membranes associated with their formation (Toyofuku et al., 2019; Toyofuku et al., 2023). The predominant type of non‐lytic BEVs produced by Gram‐negative bacteria are outer membrane vesicles (OMVs), which are formed through outer membrane blebbing and contain a specific composition (Juodeikis & Carding, 2022; McMillan & Kuehn, 2021; Toyofuku et al., 2019). In contrast, lytic BEVs are formed during cell lysis and may contain any cellular components (Toyofuku et al., 2019). Overall, BEVs can perform a wide range of functions, which can be divided into metabolism, cell interactions and resistance (Juodeikis & Carding, 2022; McMillan & Kuehn, 2021; Sartorio et al., 2021).

Determining the proteome of BEVs is a powerful tool for unravelling the molecular composition, functions and potential applications of these vesicles in various biological processes and disease contexts (Orench‐Rivera & Kuehn, 2016). The proteome of BEVs therefore provides valuable insights into the composition and functional characteristics of these vesicles. This can help identify cargo proteins such as virulence factors, enzymes and transporters or proteins involved in signalling pathways, quorum sensing or communication mechanisms. The technique allows potential enzymatic activities, nutrient acquisition abilities and other functional properties to be investigated. The approach can also be useful for identifying biomarkers of infectious diseases and other conditions. Various proteomic studies have provided evidence for the enrichment of specific proteins and the existence of specific targeting mechanisms (Orench‐Rivera & Kuehn, 2016). These have been most extensively studied in *Escherichia coli* for which the selective packaging of lipoproteins has been demonstrated (Orench‐Rivera & Kuehn, 2021). Similar work has been done in *B. thetaiotaomicron* suggesting a specific lipoprotein export sequence associated with outer membrane surface display and subsequent BEV incorporation (Valguarnera et al., 2018). However, it is important to note that the cargo protein in BEVs does not appear to be conserved across different bacteria, suggesting that vesicles produced by different organisms perform different roles (Lee et al., 2016). Additionally, the protein composition of BEVs may vary upon exposure to different environmental conditions. The lack of consistency in results also reflects different approaches in BEV isolation, which are normally extracted from bacteria cultured in rich media at widely differing times during the life cycle (Orench‐Rivera & Kuehn, 2016).

By employing various techniques, we address this latter point establishing that vesicles released during different growth stages exhibit notable variations in composition. Specifically, early vesicles are enriched in OMVs released via a specific mechanism, containing a higher proportion of lipoproteins. In contrast, lytic vesicles dominate late‐phase vesicles containing an abundance of cytoplasmic proteins. Moreover, our findings demonstrate a preferential incorporation of lipoproteins carrying a negatively charged signal peptide into OMVs. Throughout this study, we adopt the term BEVs to encompass both lytic and non‐lytic bacterial extracellular vesicles, whereas we specifically employ the term OMVs to refer to non‐lytic outer membrane vesicles.

## 2 MATERIALS AND METHODS

### 2.1 Bacterial culture conditions

*B. thetaiotaomicron* was grown in an anaerobic cabinet (10% H_2_, 5% CO_2_, 85% N_2_) at 37°C. Brain Heart Infusion (Oxoid) supplemented with 4‐μM hemin (BHIH) or Bacteroides Defined Media r6 (BDMr6) was used to culture cells. BDMr6 media is a further minimalised defined media for growing *B. thetaiotaomicron* based on our previously published media (Juodeikis et al., 2022) consisting of: 100 mM potassium phosphate pH 7.8 (KH_2_PO_4_ 9.2 mM; K_2_HPO_4_ 90.8 mM), 15 mM NaCl, 8.5 mM (NH_4_)_2_SO_4_ prepared in dH_2_O and sterilised; sterile glucose added to 30 mM and media moved to anaerobic cabinet to equilibrate for at least 24 h; after equilibration sterile components added to final concentrations: 100 μM MgCl_2_; 50 μM CaCl_2_; 2 mM L‐Cysteine hydrochloride; 10 μM FeSO_4_; during inoculation sterile components added to concentrations: 200 μM L‐methionine; 50 nm Protoporphyrin IX (prepared in 50% ethanol; 50 mM NaOH). All components purchased from Sigma‐ Aldrich unless otherwise specified.

### 2.2 Lipid quantification

FM4‐64 lipophilic fluorescent dye and linoleic acid standard has been previously utilised to quantify the amount of lipid in BEVs (Hirayama & Nakao, 2020). We adapted this approach to quantify vesicles released in culture. 20 μL of 30 μg/mL FM4‐64 (Thermo Fischer Scientific) was mixed with 180 μL of filtered culture supernatant or linoleic acid standard in water (100, 75, 50, 20, 10, 5, 1, 0 μg/mL prepared from 1 mg/mL stock) in triplicate in black 96 well plates. The samples were incubated for 10 min at 37°C and end point fluorescence was analysed using FLUOStar Omega microplate reader with pre‐set FM 4–64 settings (Excitation: 515‐15; Dichroic: auto 616.2; Emission 720‐20) utilising enhanced dynamic range. Linear standard curves were generated from the linoleic acid samples which were used to quantify the amount of lipid.

### 2.3 Nanoparticle tracking analysis

Nanoparticle quantification was carried out as previously described (Juodeikis et al., 2022). Briefly, particles were quantified using the ZetaView instrument (Particle Metrix) with ZetaView (version 8.05.12 SP1) software running a 2 cycle 11 position high frame rate analysis at 25°C. Camera control settings: 80 Sensitivity; 30 Frame Rate; 100 Shutter. Post‐acquisition parameters: 20 Min Brightness; 2000 Max Area; 5 Min Area; 30 Tracelength; 5 nm/Class; 64 Classes/Decade.

### 2.4 Cell size

To measure cell size, bacterial cells were fixed in triplicate by mixing 200 μL of culture with 200 μL 8% paraformaldehyde in double concentration PBS and incubated at ambient temperature for 15 min. The cells were then centrifuged at 8000 × *g* and washed in 1 mL of PBS twice and resuspended in 10 μL of PBS. 2 μL of the resuspended cells were spotted on 2% agarose beads prepared in 0.22 μm filtered PBS and covered with a #1.5 coverslip. Five images per replicate were collected using phase contrast microscopy using Zeiss Axio Imager M2 instrument at 630x magnification. All collected images were analysed using MicrobeJ (Ducret et al., 2016) plugin for Fiji with settings as follows: Exclude on Edges; Shape descriptors; Intensity options and Area: 0.6‐max; Length: 0.5‐max; Width: 0–1.1; Circularity: 0‐max; Curvature: 0‐max; Sinuosity: 0‐max; Angularity: 0‐max; Solidity: 0.95‐max; Intensity: 0–1550.

### 2.5 Phage plaque assay

200 μL overnight *B. thetaiotaomicron* culture was used to inoculate 10 mL Bacteroides Phage Recovery Medium (BPRM) and grown to OD_600_ ∼ 0.4 in an anaerobic cabinet (10% H_2_, 5% CO_2_, 85% N_2_) at 37°C. 200 μL of this culture and 100 μL of test supernatant was mixed into 5 mL BPRM semi‐solid agar (0.5%; 45°C) in triplicate. This mixture was plated on BPRM solid agar plates and incubated overnight in an anaerobic cabinet (10% H_2_, 5% CO_2_, 85% N_2_) at 37°C. Visual inspection of the plates was used to evaluate plaque formation.

### 2.6 BEV purification

BEVs were prepared using a further optimised purification method based on our previously described method (Juodeikis et al., 2022). Cell cultures were centrifuged at 6600 × *g* for 1 h at ambient temperature. Immediately, the supernatant was vacuum filtered using a 0.22 μm filter to ensure cell removal. The BEV containing supernatants were concentrated approximately 100‐fold using crossflow filtration (Vivaflow 50R; 100 kDa) followed by washing 5 times with 100 mL PBS, concentrating to the same volume between washes. The vesicles were then filter sterilised using a 0.22 μm syringe filter and concentrated using centrifugal concentrator (Vivaspin 20; 100 kDa; PES (Sartorius)). Vesicles were further purified using qEVsingle 35 nm column (Izon Science) as per manufacturers guidelines confirming that all particles were eluted in the expected fractions. These fractions were then pooled, concentrated using centrifugal concentrator (Vivaspin 6; 100 kDa; PES (Sartorius)) and filter sterilised using a 0.22 μm syringe filter.

### 2.7 BEV DNA protection assay

BEV associated DNA was quantified using Qubit dsDNA HS kit and Qubit 4 Fluorometer (Thermo Fischer Scientific). The vesicle samples were then treated with benzonase (Sigma‐Aldrich) and quantified again to estimate the amount of exposed DNA. The treated samples were then heated at 100°C for 30 min to lyse the vesicles, allowed to cool to ambient temperature and repeatedly treated with benzonase. The difference between heated benzonase treated and untreated samples is the estimated vesicular DNA.

### 2.8 DNA extraction and sequence analysis

DNA purification was carried out utilising 20 μL of purified BEV sample using Soil DNA extraction kit (MP Biomedicals) following the recommended protocol with adjusted buffer volumes: 80 μL SPB; 20 μL MT; 25 μL PPS; 200 μL binding matrix; eluted in 20 μL.

Fragment sizing was done using a D5000 ScreenTape (Agilent) using the Agilent Tapestation 4200 instrument. Illumina whole genome sequencing was carried out using a 20‐fold reduced volume DNA Flex prep method and sequenced paired end 150 bp using the NextSeq 500 instrument.

### 2.9 Protein analysis

Protein quantification was carried out using Qubit Protein Assay. For comparative proteomic analysis, purified BEVs in PBS were precipitated with acetone (Nickerson & Doucette, 2020) and the protein pellets were resuspended in 100 μL of 2.5% sodium deoxycholate (SDC; Merck) in 0.2 M EPPS‐buffer (Merck), pH 8, and vortexed under heating. Protein concentration was estimated using a BCA assay and approx. 100 μg of protein per sample was reduced, alkylated and digested with trypsin in the SDC buffer according to standard procedures.

After the digest, the SDC was precipitated by adjusting to 0.2% trifluoroacetic acid (TFA), and the clear supernatant subjected to C18 SPE (Reprosil, Dr. Maisch GmbH). Peptide concentration was further estimated by running an aliquot of the digests on LCMS.

Isobaric labelling was performed using a TMT 16plex kit (Thermo Fisher Scientific) according to the manufacturer's instructions with slight modifications; approx. 100 μg of the dried peptides were dissolved in 90 μL of 0.2 M EPPS buffer (Merck)/10% acetonitrile, and 250 μg TMT reagent dissolved in 22 μL of acetonitrile was added. Samples were assigned to the TMT channels in an alternating order to reduce channel leakage between different samples (Brenes et al., 2019). After 2 h incubation, aliquots of 1 μL from each sample were combined in 400 μL 0.2% TFA, desalted, and analysed on the mass spectrometer (same method as for TMT, see below, but without RTS) to check labelling efficiency and estimate total sample abundances. The main sample aliquots were quenched by adding 8 μL of 5% hydroxylamine and then combined to roughly level abundances and desalted using a C18 Sep‐Pak cartridge (200 mg, Waters).

The eluted peptides were dissolved in 500 μL of 25 mM NH_4_HCO_3_ and fractionated by high pH reversed phase HPLC. Using an ACQUITY Arc Bio System (Waters), the samples were loaded to an XBridge 5 μm BEH C18 130 Å column (250 mm × 4.6 mm, Waters). Fractionation was performed with the following gradient of solvents A (water), B (acetonitrile) and C (25 mM NH_4_HCO_3_ in water) at a flow rate of 1 mL/min: solvent C was kept at 10% throughout the gradient; solvent B: 0–5 min: 5%, 5–10 min: 5%–10%, 10–80 min: 10%–45%, 80–90 min: 45%–80%, followed by 5 min at 80% B and re‐equilibration to 5% for 24 min. Fractions of 1 mL were collected and concatenated by combining fractions of similar peptide concentration to produce 17 final fractions for MS analysis.

Aliquots were analysed by nanoLC‐MS/MS on an Orbitrap Eclipse Tribrid mass spectrometer equipped with a FAIMS Pro Duo interphase coupled to an UltiMate 3000 RSLCnano LC system (Thermo Fisher Scientific). The samples were loaded onto a trap cartridge (Pepmap Neo, C18, 5um, 0.3 mm × 5 mm, Thermo Fisher Scientific) with 0.1% TFA at 15 μL min‐1 for 3 min. The trap column was then switched in‐line with the analytical column (nanoEase M/Z column, HSS C18 T3, 1.8 μm, 100 Å, 250 mm × 0.75 μm, Waters) for separation using the following gradient of solvents A (water, 0.1% formic acid) and B (80% acetonitrile, 0.1% formic acid) at a flow rate of 0.2 μL min‐1: 0–3 min 3% B (parallel to trapping); 3–10 min linear increase B to 8 %; 10–108 min increase B to 50% (curve 4); 108–113 min linear increase B to 99 %; keeping at 99% B for 3 min and re‐equilibration to 3% B.

Data were acquired with the following parameters in positive ion mode with the FAIMS device set to three compensation voltages (−35 V, −50 V, −65 V) for 1 s each: MS1/OT: resolution 120K, profile mode, mass range m/z 400–1600, AGC target 4e5, max inject time 50 ms; MS2/IT: data dependent analysis with the following parameters: 1 s cycle time Rapid mode, centroid mode, quadrupole isolation window 0.7 Da, charge states 2–5, threshold 1.9e4, CID CE = 30, AGC target 1e4, max. inject time 50 ms, dynamic exclusion 1 count for 15 s mass tolerance of 7 ppm; MS3 synchronous precursor selection (SPS): 10 SPS precursors, isolation window 0.7 Da, HCD fragmentation with CE = 50, Orbitrap Turbo TMT and TMTpro resolution 30k, AGC target 1e5, max inject time 100 ms, Real Time Search (RTS): protein database *B. thetaiotaomicron* (uniprot.org, March 2022, 7482 entries), enzyme trypsin, 1 missed cleavage, oxidation (M) as variable, carbamidomethyl (C) and TMTpro as fixed modifications, precursor tolerance 10 ppm, Xcorr = 1.4, dCn = 0.1.

The acquired raw data were processed and quantified in Proteome Discoverer 3.0 SP1 (Thermo Fisher Scientific); all mentioned tools of the following workflow are nodes of the proprietary Proteome Discoverer (PD) software. A protein FASTA database for *B. thetaiotaomicron* (uniprot.org, May 2023, 4782 entries) was imported into PD adding a reversed sequence database for decoy searches; a database for common contaminants (maxquant.org, 245 entries) was also included. The database search was performed using the incorporated search engines Comet and CHIMERYS (MSAID, Munich, Germany). The processing workflow for both engines included recalibration of MS1 spectra (RC), reporter ion quantification by most confident centroid (20 ppm). For CHIMERYS the Top N Peak Filter was used with 20 peaks per 100 Da and the inferys_2.1_fragmentation prediction model was used with fragment tolerance of 0.6 Da, enzyme trypsin with 1 missed cleavage, variable modification oxidation (M), fixed modifications carbamidomethyl (C) and TMT16plex on N‐terminus and K. For Comet the version 2019.01 rev. 0 parameter file was used with default settings except precursor tolerance set to 6 ppm and trypsin missed cleavages set to 1. Modifications were the same as for CHIMERYS. Evaluation of the search results was performed using the Percolator node based on the *q*‐values. Identifications were calculated for False Discovery Rate (FDR) 0.01 (strict) and 0.05 (relaxed).

The consensus workflow in the PD software included the following parameters: assigning the 4 replicates/channels as described above per condition, only unique peptides (protein groups) for quantification, intensity‐based abundance, TMT channel correction values applied (WB314804), co‐isolation/SPS matches thresholds 50%/70%, normalised CHIMERYS Coefficient Threshold 0.8, normalisation on total peptide abundances, protein abundance‐based ratio calculation, missing values imputation by low abundance resampling, hypothesis testing by protein abundance based ANOVA; the adjusted *p*‐value is calculated in PD using the Benjamini‐Hochberg method. The results were exported into a table including data for normalised and un‐normalised abundances, ratios for conditions 2/3/4 versus condition 1, the corresponding *p*‐values and adjusted *p*‐values, number of unique peptides, *q*‐values and PEP‐values from Percolator, CHIMERYS and Comet identification scores, FDR confidence combined for both search engines filtered for high confidence (strict FDR 0.01) only (Table S1). Further filtering included removal of contaminants and single unique peptide matches.

All the mass spectrometry proteomics data has been deposited to the ProteomeXchange Consortium via the PRIDE partner repository with the dataset identifier PXD043651 (Perez‐Riverol et al., 2022).

Normalised abundance values of the filtered data were used to carry out principal component analysis (PCA) using R. The data were standardised by subtracting the mean and dividing by the standard deviation followed by the computation of the correlation matrix and PCA analysis (Supplementary Text 1).

### 2.10 Cryo‐TEM sample preparation and analysis

BEV sample was prepared on Quantifoil R2/2 300 mesh copper grids (Agar Scientific) that were glow discharged using air for 60 s at 8 mA in an Ace 200 (Leica Microsystems). 3.5 μL of sample was applied to the grid within the chamber of a Vitrobot IV (Thermo Fisher Scientific) set to 4°C, 100% relative humidity, 10 s ‘Wait Time’, blot time of 2 or 2.5 s, blot force 8 and frozen in liquid ethane.

Grids were imaged using a Talos F200C transmission electron microscope (Thermo Fisher Scientific) operated at 200 kV, equipped with a 4k OneView CMOS detector (Gatan). Automated data acquisition was setup using EPU v2.12.1 (Thermo Fisher Scientific), each image had a 1 s exposure with a sample dose of 40 e^−^/ Å^2^, defocus of −2.5 μm, nominal magnification of 57,000x and a calculated pixel size of 2.8 Å.

Vesicle measurements were carried out in Fiji by measuring a circular area covering the whole vesicle (Schindelin et al., 2012). The diameter was calculated using the measured area. At least 2000 vesicles were measured per sample. To remove bias, all visible vesicles were measured in the analysed images.

### 2.11 NMR metabolite analysis

A minimum of 100 μL of filtered cultures were mixed with phosphate buffer (NaH_2_PO_4_ (21.7 mM), K_2_HPO_4_ (82.7 mM), NaN_3_ (8.6 mM), 3‐(methylsilyl)‐propionate‐*d*_4_ (TMSP, 1.0 mM) in D_2_O) (Vignoli et al., 2019) directly in an NMR tube (Wilmad 528‐PP‐7, 5 mm). The spectra were recorded on a Bruker Avance III 500 MHz spectrometer, equipped with an inverse triple resonance *z*‐gradient probe. All ^1^H NMR spectra were acquired using a minimum of 256 scans, a spectral width of 7500 Hz, acquisition time of 2.36 s, recycle delay of 4 s and a ^1^H π/2 radiofrequency (*rf*) pulse of 10 μs, using a combination of Perfect Echo and WATERGATE pulse sequences (‘*PEW5*’) (Adams et al., 2013) for suppression of *J* modulation and highly selective solvent suppression, giving narrow suppression bands. All spectra were acquired at 25°C and in triplicate per time point. Metabolites of interest were quantified using the NMR software suite v7.6 Profiler (Chenomx).

### 2.12 Construction and transformation of TargetSeq plasmids

To generate Nanoluciferase plasmids containing the different TargetSeq (Table S2) peptides we used the *B. thetaiotaomicron* VPI‐5482 integrative plasmid pNBU2_erm‐TetR‐P1T_DP‐GH023 (Lim et al., 2017) as the backbone. Firstly, the tetracycline expression cassette (AflII/BamHI) was replaced with a modified BT_1830 promoter together with RBS7 (Whitaker et al., 2017) encoding for a Nanoluciferase with an N‐terminal signal sequence from BT_3742 (32 amino acids) linked by a TEV protease site. This fragment was flanked by two synthetic terminators (pIBATH.51). The N‐terminal signal sequence was then modified to allow for simple insertion of short DNA fragments (overlapped primers with a 3′ GA overhang) to exchange the TargetSeq peptide without affecting upstream or downstream sequence (pIBATH.TA). Overlapping primers were then used to generate plasmids containing the different TargetSeq sequences. All cloning was carried out using *E. coli* PIR1 cells (Thermo Fischer Scientific). The constructed plasmids were used to transform *B. thetaiotaomicron* VPI‐5482 using triparental mating using *E. coli* PIR1 carrying the target plasmid as the donor strains and *E. coli* pRK2013 (Clontech) as the helper strain. Cells were plated on BHIH agar containing 200 μg/mL gentamicin and 25 μg/mL erythromycin.

### 2.13 NanoLuciferase assay

Two 96 well plates were set up with 200 μL cultures in triplicate for each of the tested constructs with starting optical density at 600 nm of 1. One plate was analysed 5 h post inoculation while the second plate was analysed 24 h post inoculation. 5 μL of culture or supernatant was mixed with 45 μL of PBS and 50 μL Nano‐Glo Luciferase Assay System (Promega) prepared substrate in white 96 well plates and analysed using FLUOStar Omega microplate reader with pre‐set Nanoluciferase settings (Emission 470–80).

## 3 RESULTS

### 3.1 Limitations of BEV analysis in rich growth media

**FIGURE 1:**
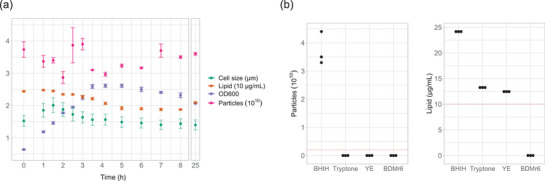
Particle and lipid analysis highlighting limitations of using complex media in BEV studies. (a) *B. thetaiotaomicron* growth analysis in commonly used Brain–Heart Infusion media supplemented with hemin (BHIH). FM4‐64 and particle count signal does not correlate with growth. Size of fixed non‐dividing cells measured using light microscopy; lipid content of supernatants measured using FM4‐64 lipophilic dye; OD600 – optical density of cultures determined at 600 nm; particle numbers determined using ZetaView nanoparticle tracking analysis instrument. Experiment carried out in triplicate. (b) Particle count and FM4‐64 analysis of (BHIH), tryptone (10 g/L), yeast extract (YE; 5 g/L) and our media (BDMr6). Red dotted line represents detection limit. Analysis carried out on three separately prepared aliquots from individual powder stocks, since these represent technical replicates no formal statistical analysis is performed. BHIH, Brain–Heart Infusion media supplemented with hemin.

To define the parameters of BEV formation in *B. thetaiotaomicron*, we conducted a time‐dependent analysis of vesicle release. We set up triplicate cultures in Brain–Heart Infusion media supplemented with hemin (BHIH) and monitored culture optical density, fixed cell size, particle count and supernatant lipid content (Figure 1a). High levels of lipid, and particles were evident at inoculation (0 h). At first, we hypothesised that these vesicles could be remnants from the inoculum or released during the inoculation process as a result of cell lysis. However, upon testing three separate sterile batches of BHIH, we observed the presence of these particles in the media (Figure 1b). The hydrodynamic diameter of these particles was also similar (media: 111.00 ± 0.41 nm; time 0 h: 111.33 ± 0.47), further supporting that the particles observed at 0 h were particles present in the media. Additionally, significant shifts in particle hydrodynamic diameter were only observed after overnight incubation when the diameter of the particles increased to 139.33 ± 1.0 nm (*t* = 25 h). We also investigated other commonly used media components, namely tryptone (10 g/L) and yeast extract (5 g/L) for the presence of particles, although none were detected. However, the more sensitive lipid assay detected the presence of lipid in both media components. Notably, complex media components have high batch to batch and supplier variation as such the presented results should be considered as descriptive and not quantitative for the specific components. The findings from this analysis highlight the need to avoid complex media components in vesicle analysis due to the presence of contaminating particles and lipids. This is particularly relevant for studies investigating BEV effects on cells or those characterising chemical composition. Consequently, to examine the dynamics of vesicle release, we formulated a chemically defined media for the growth of *B. thetaiotaomicron* free of animal‐derived products. This media was developed by building on our previously reported media and is referred to as BDMr6 (Juodeikis et al., 2022). In comparing the concentration of particles and lipid in this media with BHIH, yeast extract and tryptone, BDMr6 contained no detectable particles (Figure 1b). This media was utilised in all further analyses of *B. thetaiotaomicron* BEV release dynamics.

### 3.2 *B. thetaiotaomicron* BEV release dynamics in chemically defined media

**FIGURE 2:**
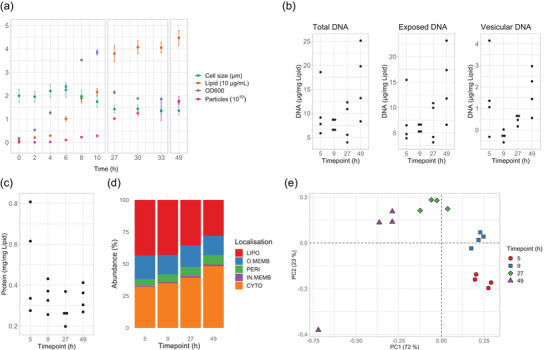
Analysis of *B. thetaiotomicron* BEVs produced in chemically defined media. (a) *B. thetaiotaomicron* growth analysis in BDMr6. The size of fixed non‐dividing cell size was measured using light microscopy; The lipid content of the supernatant was measured using FM4‐64 lipophilic dye; Optical density of cultures was measured at 600 nm (OD600); Particle number was determined using the ZetaView nanoparticle tracking analysis instrument. (b) Analysis of BEV associated double stranded DNA adjusted to the amount of lipid. Differences between time points are not statistically significant except for vesicular DNA between 9 and 49 h (1‐way ANOVA following Dunnett correction excluding the 5 h time point *p* = 0.0023). (c) Total protein present in BEVs adjusted to the amount of lipid. (d) Localisation of BEV proteins at 4 different timepoints. Predicted protein localisation. (e) Principal component analysis analysis of BEV proteomes over time. BEV, bacterial extracellular vesicles; CYTO, cytoplasmic; IN.MEMB, inner membrane; LIPO, lipoproteins; O.MEMB, outer membrane; PERI, periplasmic.

To investigate if *B. thetaiotaomicron* produced different types of BEVs we compared vesicles harvested at four different timepoints using BDMr6 media and end‐point cultures. For the first two timepoints, the culture volumes were doubled and split between two bottles to ensure sufficient material was generated for analysis. Initial attempts to establish these cultures in larger volumes (changing from 500 to 1000 mL in representative Duran bottles) proved unreliable as bacterial growth was significantly affected by different sized culture bottles most likely due to changes in gas exchange rate. To ensure rapid growth, *B. thetaiotaomicron* cell pellets from 50 mL BDMr6 cultures were used to inoculate the replicates. As previously, we monitored four characteristics of growth with OD_600_ monitored in all cultures and the other characteristics analysed for samples harvested at 49 h (Figure 2a). Growth dynamics showed a slow release of BEVs (Particles; Lipid) for 4–6 h, after which a significant increase in release of BEVs was observed until the peak OD_600_ value was reached at around 10 h post inoculation. Thereafter, the OD_600_ values exhibited an unexpected and substantial decline alongside continued vesicle release. To investigate the possibility of contaminating bacteriophage, we conducted experiments using fresh bacterial stocks, which yielded similar results, and phage plaque assays using the supernatant from overnight cultures, which yielded negative results, ruling out the presence of contaminating bacteriophage as the cause.

**FIGURE 3:**
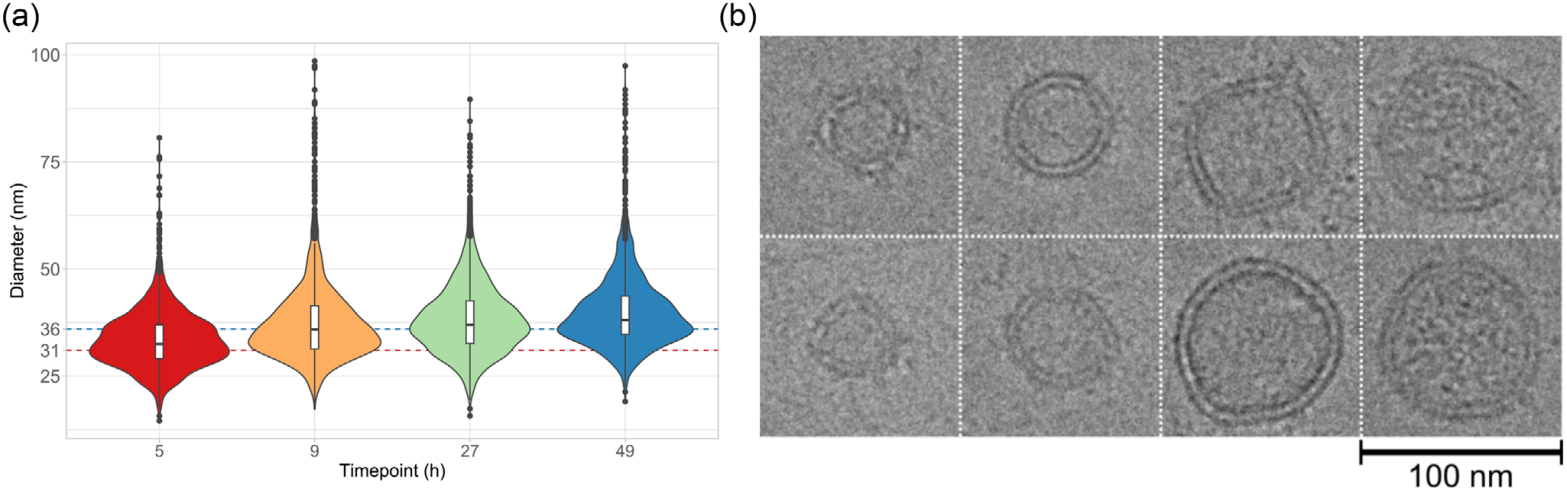
Cryo‐TEM analysis of *B. thetaiotaomicron* BEVs. (a) Combined violin and box plots of BEV size distribution analysed using Cryo‐TEM. Cut‐off value = 100 nm; *n* > 2000. Dashed lines show peak values for 5‐ and 49‐h samples. (b) Representative Cryo‐TEM micrographs of different sized BEV particles observed. Particles shown in the far right column display increased electron density. BEV, bacterial extracellular vesicles.

BEVs were purified using cross filtration followed by size exclusion from cultures harvested at four different timepoints: 5‐, 9‐, 27‐ and 49‐h post inoculation representing half‐peak OD_600_, peak OD_600_, stationary phase and extended stationary phase. DNA (Figure 2b) and protein (Figure 2c and d; Table S1) content was determined in parallel with nanoparticle tracking analysis and Cryo‐TEM (Figure 3, Figures S2 and S3).

DNA was co‐purified with BEVs (Figure 2b “Total DNA”). Upon treatment with Benzonase nuclease, the level of DNA was reduced in the samples, indicating that the majority of the DNA was externally exposed rather than residing internally within the lumen of BEVs, where it would be shielded from nucleases (Figure 2b “Exposed DNA”). To obtain a more precise assessment of DNA within vesicles, Benzonase‐treated vesicles were subjected to lysis through heating at 100°C for 30 min, followed by repeat DNA analysis. By comparing the values obtained before and after nuclease treatment the quantity of DNA residing within the lumen of *B. thetaiotaomicron* BEVs could be estimated (Figure 2b “Protected DNA”). This approach allowed to rule out nonspecific signal; however, this also resulted in some negative values. There is some suggestion of a progressive increase in DNA presence both internally and externally to the vesicles over time, with the difference between vesicular DNA between 9 and 49 h being statistically significant (*p* = 0.0023, 1‐way ANOVA followed by Dunnett correction). However, due to small sample size and large variation further analysis with increased sample size is required to confirm this finding.

The size of DNA fragments extracted from BEV preparations determined using the ScreenTape system revealed sizes ranging from 1 to 10+ kb with a peak at around 6 kb. Illumina whole genome sequencing revealed the presence of both genomic and plasmid DNA in all samples in the absence of any region‐specific enrichment.

Taken together, the results indicate that the majority of DNA content in the analysed BEV preparations of *B. thetaiotaomicron* is derived from extracellular DNA released during cell lysis, with a minor fraction present in lytic vesicles released during the late stationary phase. The high variability of DNA levels observed at 5 h are likely due to the carry‐over of vesicles from the inoculation process, coupled with the cellular transition to the exponential growth phase (Figure 2b “Total DNA”). During inoculation, cellular metabolism is comparable to that of late‐phase cells, and vesicles released during the transition to the exponential phase resemble those of late‐phase BEVs.

There was no evidence for a difference in BEV protein to lipid ratio over time, after the initial 5 h time point (Figure 2c). The protein composition of purified BEVs was investigated using time resolved comparative proteomics (Figure 2d and 2e; Table S1). Collected data is available via ProteomeXchange with identifier PXD043651 (Perez‐Riverol et al., 2022). As part of this analysis proteins were categorised according to their predicted cellular localisation and the relative abundance was followed over time (Figure 2d; localisation predicted using SignalP 6.0 [Teufel et al., 2022]). Principal component analysis (PCA) was used to highlight patterns in the relative abundances of identified proteins amongst the 16 samples (Figure 2e). In PCA the BEV proteome changes over time were captured on PC1 and PC2, accounting for 72% and 23% variance, respectively. Together these show that the time‐dependent changes are primarily driven by the changes in the relative abundances of lipoproteins and cytoplasmic proteins. One possible explanation for this observation is the variability in the types of vesicles produced with vesicles released at earlier timepoints being OMVs containing specific proteins, whereas BEVs released at later timepoints are predominantly lytic vesicles containing non‐selective proteins. This would also explain increased levels of DNA observed in BEVs at the later timepoints. Notably, one replicate collected at 49 h did not cluster; however, the sample still followed the trend on the major PC1 axis accounting for 72% of the observed variance.

Differences were also observed in vesicle size by Cryo‐TEM (Figure 3; Figure S1) with a shift towards larger vesicles, suggesting that OMVs produced at earlier timepoints are smaller than lytic vesicles produced later. Overall, vesicles of varying size were coincident with vesicles of increased electron density (Figure 3b). As noted previously, a variety of different vesicle morphologies were evident, including those with multiple membranes as well as vesicles containing smaller vesicles within them although these were not present in significant amounts (<0.1 %) (Toyofuku et al., 2019). Notably, no significant differences were observed in the hydrodynamic diameters of BEVs produced at different timepoints.

**FIGURE 4:**
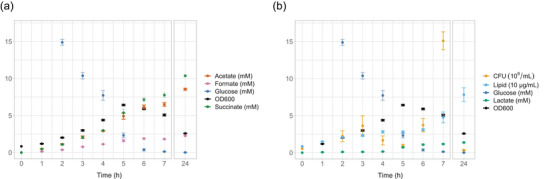
*B. thetaiotaomicron* metabolite, BEV and CFU analysis. (a) Graph highlighting the major fermentation products produced by *B. thetaiotaomicron* in BDMr6 with glucose as the carbon source. Mixed acid fermentation at 2:2:1 ratios of succinate:acetate:formate released by the cells. OD600, optical density of culture at 600 nm. Error bars shows one standard deviation. Experiment carried out in triplicate. (b) Graph highlighting change in CFU in relation to OD600, vesicle lipid content (FM4‐64) and lactate. Lipid content of supernatant measured using FM4‐64 lipophilic dye. Early glucose timepoints, ethanol and propionate analysis can be seen in Figure S2. BEV, bacterial extracellular vesicles; CFU, colony forming unit.

To further understand culture growth dynamics and how these relate to BEV release, colony forming unit (CFU) determinations and NMR based culture metabolite analysis was carried out (Figure 4 and Figure S2). The starting OD_600_ for this experiment was higher in order to capture the reduction in OD_600_ after the peak OD_600_ is reached. In general, *B. thetaiotaomicron* utilised mixed acid fermentation with equimolar release of acetate and succinate being the major products (Figure 4a). Additionally, formate was released early followed by a switch to lactate at approximately 4 h post‐inoculation (Figure 4). Small amounts of propionate and ethanol were also detected after overnight culture (Figure S2). Notably, the ethanol detected at early timepoints was due to the addition of protoporphyrin IX, which is prepared in ethanol. It was not possible to calculate the carbon balance as the glucose was removed from the supernatant faster than the appearance of the acids. These findings suggest two possibilities: either a significant portion of glucose is converted to carbon dioxide, or glucose is stored within the cells. It is worth mentioning that *B. thetaiotaomicron* possesses a potential glycogen synthase (BT_4307; Q89ZR9), indicating the potential for glycogen storage. Additionally, the continuous release of acids even after the depletion of glucose from the media (at 6 h post‐inoculation) further supports the notion of intracellular glucose storage.

CFU analysis revealed a notable drop at around 4 h post‐inoculation in the absence of any decrease in OD_600_. Although cell size analysis was not carried out for this experiment, the half peak OD_600_ correlated with the size decrease seen in the initial analysis (Figure 2a). The decrease in CFU observed between 4 and 6 h coincided with the depletion of glucose and the release of lactate, suggesting a metabolic shift. Interestingly, the release of vesicles, as quantified by the amount of lipid, appeared to cease during this metabolic transition (Figure 4b). Surprisingly, despite the decline in optical density at OD_600_, indicating a decrease in cell density, the CFU counts continued to rise. This indicates that the number of viable cells is not accurately reflected by CFU analysis alone. Moreover, as the OD_600_ started to decrease, the CFU counts increased concurrently with the ongoing release of vesicles. Overnight growth led to a drop in CFUs and further release of vesicles. Overall, the observed culture dynamics support the view of differential BEV release, with the initial release of vesicles occurring before the culture reaches half peak OD600 when a metabolic shift is observed. This is followed by a lag in vesicle release while the cells reach peak OD600, and lactate is produced. The subsequent decrease in OD600 coincides with the resumption of vesicle release.

### 3.3 Analysis of *B. thetaiotaomicron* BEV targeting sequence

**FIGURE 5:**
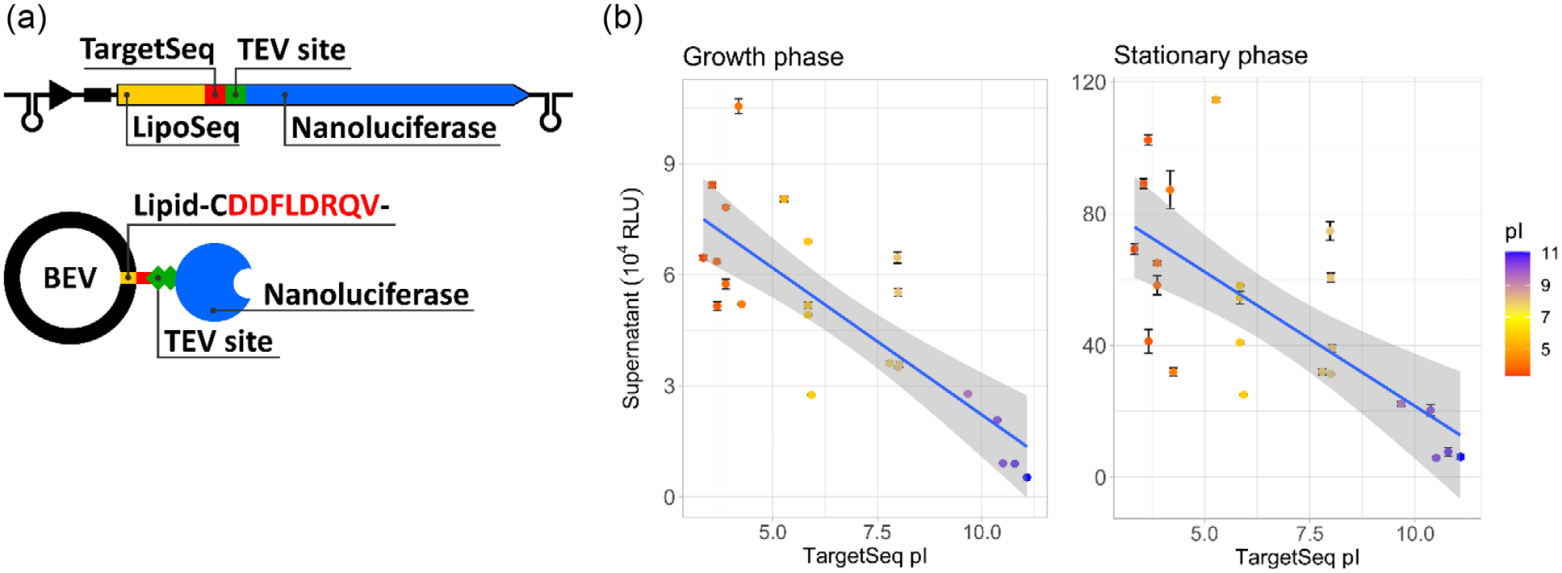
BEV incorporation signal peptide analysis. (a) Schematic of Nanoluciferase reporter construct used to evaluate targeting peptide sequences. Upper section depicts the genetic constructs with lower section depicting the expected protein topology on BEVs. LipoSeq is BT_3742 Sec targeting sequence which is cleaved and lipidated at a cysteine (C) residue; TargetSeq (red) is the variable 8 amino acid region which defines the pI (see Table S2 for all sequences); TEV site is the TEV protease cleavage site. (b) Graphs show Nanoluciferase signal in the supernatant of cultures two different growth phases plotted against the isoelectric point (pI) of the TargetSeq. Error bars represent one standard deviation between triplicate samples. BEV, bacterial extracellular vesicles.

Proteins enriched in *B. thetaiotaomicron* vesicles have been suggested to carry a specific lipoprotein export sequence (LES) motif CS(D/E)_2_ (Valguarnera et al., 2018). However, in our proteomics analysis some of the highly enriched proteins did not carry this motif (Table S1). To investigate this further a Nanoluciferase based reporter system was developed to evaluate lipoprotein incorporation into BEVs (Figure 5a). The reporter constructs consisted of a lipoprotein export sequence followed by a variable region encoding 8 amino acids, a TEV protease cleavage site and Nanoluciferase. The coding sequence was expressed using a constitutive promoter and integrated into the *B. thetaiotaomicron* genome using a single copy integration vector. This system allowed to test the hypothesis that incorporation of proteins into vesicles is charge depended.

Twenty‐four naturally occurring sequences from *B. thetaiotaomicron* lipoproteins representing a range of isoelectric points covering the 8 amino acids post lipidated cysteine were used in this study (Table S2). A cytoplasmic version of the protein was also included as a control. The Nanoluciferase signal from these constructs was quantified for individual cultures (in triplicate) and their supernatants at 5‐ and 24‐h post‐inoculation representing growth and stationary phase (Figure 5b and Figure S3). As illustrated in Figure S3a, the overall signal variation was independent of the isoelectric point (pI) of the target sequences. A pre‐optimisation experiment confirmed that the signal detected in the supernatant was attributed to Nanoluciferase bound to BEVs. Using a protein concentrating column with a molecular weight cut‐off of 100 kDa over 99% of the Nanoluciferase signal was observed in the retentate, in contrast to an equivalent signal detected in both the retentate and flow‐through when wild‐type BEVs were mixed with soluble Nanoluciferase.

Overall, a negative trend of Nanoluciferase incorporation into BEVs was seen based on the pI of the tested sequences. This suggests that negatively charged amino acids found C‐terminally of the post‐lipidated cysteine encourages protein incorporation into BEVs. Notably, higher BEV localisation for the proposed CS(D/E)_2_ BEV enrichment sequence was not seen when compared to a different sequence with a similar pI (TargetSeq_BT_3742_: ‐CSDDDDVKY‐; pI = 3.66; 6 × 10^4^ and 76 × 10^4^ RLU respectively; compared to TargetSeq_BT_3745_: ‐CIQDEALNS‐; pI = 3.66; 8 × 10^4^ and 79 × 10^4^ RLU respectively). One outlier was observed which showed significantly higher export and was excluded from Figure 5b (TargetSeq_BT_1762_: ‐CDDFLDRQV‐; pI = 3.8; 80 × 10^4^ and 213 × 10^4^ RLU respectively). When cytoplasmic Nanoluciferase was expressed, no luminescence signal was detected in the supernatants harvested during the growth phase, with only a low signal detected at stationary phase (3.7 × 10^4^ RLU), which is likely due to cell lysis. During the growth phase, a stronger correlation was observed, which could potentially be attributed to a lower abundance of lytic vesicles at that timepoint.

These findings suggest that the overall amino acid charge of the target sequence, rather than specific individual amino acids, provides a better prediction of protein incorporation into BEVs. It is important to note that the previously described CS(D/E)_2_ sequence is predictive of protein incorporation into BEVs due to its low pI, although it does not capture the full range of preferentially incorporated proteins. Additionally, negatively charged amino acids located C‐terminally of the post lipidated cysteine in lipoproteins have been shown to be indicative of surface display in closely related *Bacteroides* species suggesting that these enriched lipoproteins are exposed on the surface (Lauber et al., 2016). Collectively, these observations indicate that surface‐exposed lipoproteins are enriched in OMVs. However, it is important to note that these proteins are not exclusively produced for OMV incorporation, as only a subset of them are packaged into OMVs (Figure S3b). This suggests that OMVs serve as an extension of the outer membrane, facilitating display of lipoproteins.

### 3.4 Theoretical *B. thetaiotaomicron* BEV proteome

Assuming the pI of the target sequence is indicative of BEV lipoprotein enrichment in *B. thetaiotaomicron*, we can therefore predict all enriched proteins independent of growth conditions.

SignalP 6.0 (Teufel et al., 2022) analysis predicted the presence of 729 lipoproteins in *B. thetaiotaomicron* VPI‐5482 (Table S3). These lipoproteins make up approximately 15.2% of the proteome. In comparison, the model organism *E. coli* K12 is predicted to have 114 lipoproteins, accounting for 2.6% of its proteome. Further analysis was conducted on the pI (Kozlowski, 2016) of the target sequences of the 729 predicted lipoproteins in *B. thetaiotaomicron*. Among these lipoproteins, 468 were identified with a target sequence pI below 5.2, indicating that these proteins are likely to be preferentially enriched in BEVs.

Of the 468 lipoproteins identified, 288 were associated with polysaccharide metabolism and were localised within polysaccharide utilisation loci (PUL) according to the CAZy database. For the remaining 180 proteins, we examined their InterPro annotations to identify common functionalities. Among these proteins, 120 had InterPro annotations, and of these, 150 annotations were unique (Table S3). These annotations were then categorised based on related functionalities, leading to the identification of putative BEV‐associated proteases, nucleases, proteins involved in nutrient binding, adhesion and bacterial resistance (Table 1 and Table S4). It is worth noting that multiple annotations had no known function with 60/180 proteins identified lacking any InterPro annotations.

**TABLE 1:** Unique InterPro annotations found in predicted *B. thetaiotaomicron* BEV enriched lipoproteins together with functional predictions based on annotations and/or genetic locus.

InterPro annotations	Functional prediction
IPR029045/IPR005151/IPR041613/IPR028204/IPR001478/IPR036034/ IPR041489/IPR008969/IPR006260/IPR037682/IPR025896/IPR000200/ IPR038765/IPR044934/IPR005077/IPR023852/IPR024079/IPR008754/ IPR008915	Protease and associated
IPR001604/IPR020821/IPR044929/IPR044925/IPR040255/IPR045939	Endonuclease
IPR029052/IPR004843/IPR019079	Metallophosphatase
IPR017946	Phospholipase
IPR017853/IPR011050/IPR008928/IPR013780/IPR012341/IPR002241/ IPR041233/IPR025112/IPR038653/IPR024361/IPR013785/IPR029058	Carbohydrate metabolism
IPR011044/IPR043780/IPR011048/IPR036941/IPR015943	Cobamide uptake locus proteins
IPR006059/IPR001188/IPR025921	Nutrient binding
IPR025049/IPR042278/IPR029140/IPR029141/IPR014941	Fimbriae associated
IPR003343/IPR008979	Adhesion
IPR038143/IPR035376/IPR038179/IPR024299/IPR028967/IPR009056/ IPR036909/IPR010538/IPR001279/IPR036866/IPR014867/IPR036505/ IPR002502/IPR006619/IPR015510	Immunity/resistance

Abbreviation: BEV, bacterial extracellular vesicle.

## 4 DISCUSSION

**FIGURE 6:**
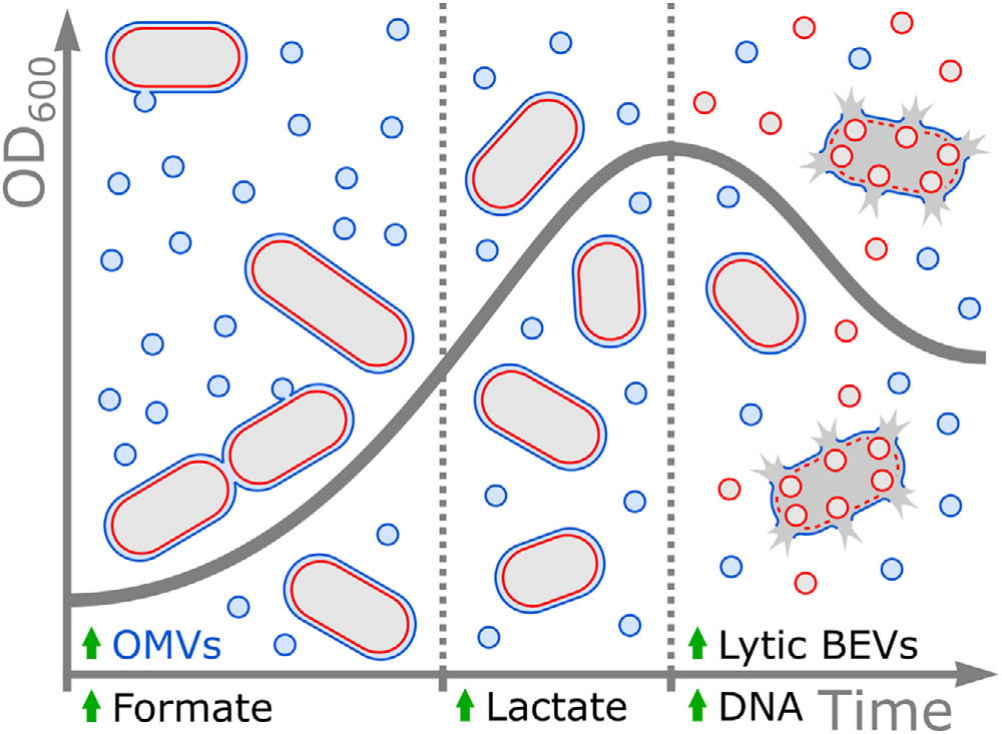
Proposed *B. thetaiotaomicron* BEV release dynamics. BEV release in BDMr6 occurs in three phases. The first phase is defined by an increase in cell size and release of specialised OMV and formate. During the second phase, lactate is released, cells reduce in size with a reduction in OMV production. The final phase is defined by a decrease in cell density and release of DNA and lytic BEVs. BEV, bacterial extracellular vesicles; OMV, outer membrane vesicles.

This study highlights the importance of considering media contaminants when studying BEVs. Various components of complex media, such as particles and lipids, can have an impact on downstream processing. To minimise these effects, chemically defined media that is free of vesicle contaminants should be used. Alternatively, if rich media is necessary, vesicles should be depleted prior to use, as recently proposed (Le et al., 2023). By utilising a chemically defined media (BDMr6) this study demonstrates that *B. thetaiotaomicron* releases distinct sub‐populations of vesicles. Initially released vesicles are enriched in non‐lytic OMVs containing specific lipoproteins, while the lytic vesicles released later carry non‐selective cargo (Figure 6). Furthermore, the study provides new insights into the lipoproteome of *B. thetaiotaomicron* OMVs.

A major difficulty in interpreting data using BEV preparations is vesicle heterogeneity and the presence of lytic and non‐lytic vesicles. Using *B. thetaiotaomicron* we confirm the presence of different types of vesicles produced at different stages of their growth. A limitation of these experiments is the persistence and carryover of vesicles produced at earlier stages of growth into later timepoints and as such, BEV preparations invariably contain a mixture of old and new vesicles. Additionally, the purification method used involves multiple wash steps which may remove proteins weakly associated with BEVs. A proportion of initially released vesicles will therefore originate from older cells used to initiate new cultures and will be carried over and contaminate vesicle preparations. When cultured in BDMr6 *B. thetaiotaomicron* release BEVs during two different phases of their growth. The initial release of non‐lytic OMVs occurs before a metabolic shift marked by the release of lactate (Figure 4). Notably, this occurred in the absence of a shift in pH, as the media contains 100 mM phosphate which provides a large buffering capacity (data not shown). The second phase of BEV release occurs upon completion of the metabolic shift as indicated by a rapid increase in CFUs with OD_600_ providing a better estimate of cell numbers compared to CFUs during the metabolic shift. This is evident as CFU counts decrease, indicating that not all growing cells are recovered. However, CFUs remain a more reliable indicator of the bacterial metabolic state. Considering the observations made during the reduction in OD_600_, the vesicles detected are expected to be predominantly lytic vesicles. This coincides with the depletion of extracellular glucose and the culture transitioning into the stationary phase. Some cells may undergo lysis, leading to the release of lytic BEVs and DNA, potentially forming a protective matrix. Meanwhile, other cells may transition into robust and adaptable forms, as evidenced by their high CFU recovery. These differential phases may represent *B. thetaiotaomicron* adaptation to different nutrient availability within the gastrointestinal tract. We have previously reported that BEVs carrying specialised proteins involved in nutrient metabolism are released in parallel with increased nutrient availability after a meal is consumed (Stentz et al., 2022). Once the nutrients are depleted the bacteria need to adapt to the new conditions, at which point lytic BEVs may be released which may play a role in adaptation and long‐term survival. It is worth noting that the observed vesicles exhibited a narrow size range, with only a few exhibiting a double membrane. These findings suggest that the production of lytic vesicles is a tightly regulated process. Previous work demonstrated that prophage dependent lysis is involved in lytic BEV release; however, we were unable to identify a homologous system in *B. thetaiotaomicron* (Jiang et al., 2022; Turnbull et al., 2016). Notably, this does not rule out the involvement of other unique lytic prophage proteins as, based on sequence similarities, a large proportion of identified proteins have no known function.

Considerable efforts have been made to uncover the cargo carried by BEVs in *B. thetaiotaomicron* including identifying potential signal sequences that correlate with surface exposure (Lauber et al., 2016; Valguarnera et al., 2018). Notably, this correlation was stronger during the growth phase compared to late stationary phase, supporting the presence of lytic vesicles containing non‐selectively expressed proteins released during transition into stationary phase. Additionally, our findings indicate that only a portion of the produced protein is exported into BEVs (Figure S3). One possible explanation for this phenomenon is that some proteins may become “trapped” in the cytoplasm due to oversaturation of the export machinery or the time it takes for the cell to process the lipoprotein. However, neither explanation is likely to be correct considering the observed outlier (TargetSeq_BT_1762_: ‐CDDFLDRQV‐; pI = 3.8) which had overall similar protein levels with significantly higher BEV incorporation. This outlier raises the question of whether this specific sequence is selective for vesicle incorporation rather than being solely dependent on overall charge. Overall, our findings support the hypothesis that surface lipoproteins with a negatively charged TargetSeq are enriched in OMVs, but not lytic vesicles. A very recent study described in a pre‐print publication utilised proteomic analysis to reach the same conclusion (Sartorio et al., 2023). Furthermore, in their work they show that integral outer membrane proteins are excluded allowing surface lipoproteins, which form strong associations with these integral proteins, to be retained specifically on the outer membrane.

The idea of charge being of primary importance in targeting lipoproteins to OMVs is further supported by the analysis of the theoretical *B. thetaiotaomicron* OMV lipoproteome. Based on TargetSeq analysis, most of the proteins destined to non‐lytic vesicles are involved in carbohydrate metabolism as indicated by their presence in polysaccharide utilization loci (PUL) associated loci. These predicted lipoproteins represent a range of predicted binding proteins and hydrolases, which likely bind or degrade complex insoluble carbohydrates. We postulate that having different hydrolases displayed on BEVs would facilitate degradation of complex carbohydrates which require the cleavage of multiple different glycosidic bonds. Additionally, the presence of different binding proteins would enable BEVs to bind these complex molecules limiting diffusion while capturing any of the released soluble sugars. Notably, the study by Sartorio and colleagues describes the incorporation of various hydrolases into BEVs and, although their data shows enrichment of the binding proteins from these operons, this observation is not discussed (Sartorio et al., 2023). Thus, BEVs offer a unique solution for bacteria to increase their surface area without expanding their volume. Furthermore, BEVs can package various hydrolases and binding proteins, enabling the extracellular breakdown of a wide range of carbohydrate bonds while minimising diffusion. While soluble sugars are typically assumed to be readily available for bacterial uptake, we propose that the presence of binding proteins on BEVs allows for the capture of these sugars. Subsequently, the captured sugars are released only to bacterial species equipped with the corresponding uptake machinery, similar to our previous observations regarding BEV‐mediated cobalamin acquisition (Juodeikis et al., 2022).

In addition, our approach has allowed us to identify several other OMV enriched proteins, including three capsular polysaccharide proteins specific to locus 8 (Q8ABP3; Q8ABP5; Q8ABP6) (Porter et al., 2017). This is the only capsular polysaccharide locus shared between *B. thetaiotaomicron* and other *Bacteroides* species. Furthermore, we have detected a range of other active proteins, likely produced under specific conditions. It is worth noting that a significant proportion of the identified lipoproteins lack a predicted function. These proteins should be considered as being both bacterial and OMV displayed, with the potential to interact with their environment, including mediating interactions with the host.

## AUTHOR CONTRIBUTIONS

**Rokas Juodeikis**: Conceptualisation; formal analysis; investigation; methodology; writing—original draft. **Carlo de‐Oliveira Martins**: Formal analysis; methodology. **Gerhard Saalbach**: Formal analysis; methodology. **Jake Richardson**: Formal analysis; investigation. **Todor Koev**: Formal analysis. **Marianne Defernez**: Data curation; formal analysis. **Martin Warren**: Formal analysis; funding acquisition; project administration; supervision; writing—review and editing.

## CONFLICT OF INTEREST STATEMENT

The authors report no conflict of interest.

## Supporting information

Supporting information.

Supporting information.

Supporting information.

Supporting information.

Supporting information.

## Data Availability

The authors confirm that the data supporting the findings of this study are available within the article and its supplementary materials.
