# Supplementary material for: Differential temporal release and lipoprotein loading in B. thetaiotaomicron bacterial extracellular vesicles
Source: J Extracell Vesicles. 2024 Jan 19;13(1):12406. doi: 10.1002/jev2.12406 (PMC10797578; doi:10.1002/jev2.12406)
Supplement: Supplementary file 5 — Supporting information. [file JEV2-13-12406-s003.docx]

## Title:

Differential temporal release and lipoprotein loading in *B. thetaiotaomicron* bacterial extracellular vesicles.

## Authors:

Rokas Juodeikis, Carlo Martins, Gerhard Saalbach, Jake Richardson, Todor Koev, Dave J. Baker, Marianne Defernez, Martin Warren, and Simon R. Carding

# Supplementary material

## Supplementary Table 1

Supplementary Table 1 [Proteomics].xlsx

## Supplementary Table 2

Supplementary Table 2[Target sequences].xlsx

## Supplementary Table 3

Supplementary Table 3 [LipoP analysis].xlsx

## Supplementary Table 4

Supplementary Table 4 [Additional Interpro annotations].xlsx

## Supplementary Text 1

The following R script was used to carry out PCA analysis and generate the plot:
# Install and load packages

install.packages("readxl")

install.packages("ggplot2")

library(readxl)

library(ggplot2)

PCA_data <- read_excel("PCA_data.xlsx") # Data loaded into R

numerical_data <- PCA_data[,3:18] # Non-numerical data (column 1 contains Uniprot values; Column 2 contains localisation) removed for PCA analysis

normalised_data <- scale(numerical_data) # Data is standardized by subtracting the mean and dividing by the standard deviation.

corr_matrix <- cor(normalised_data) # Compute the correlation matrix

PCA_analysis <- princomp(corr_matrix) # Conduct PCA analysis

summary(PCA_analysis) # Extract cumulative proportion information

PCA_scores <- data.frame(PCA_analysis$scores[, 1:2]) # Extract scores

PCA_scores$timepoint <- as.factor(c(5,5,5,5,9,9,9,9,27,27,27,27,49,49,49,49)) # Assign timepoint values

ggplot(PCA_scores, aes(x=Comp.1, y=Comp.2, color=timepoint)) + # PCA plot

geom_point(size=2) +

geom_hline(yintercept=0, linetype="dashed") +

geom_vline(xintercept=0, linetype="dashed") +

labs(y="PC2 (23 %)", x="PC1 (72 %)", color = "Timepoint (h)") +

scale_colour_brewer(palette = "Set1") +

theme_light() +

theme(axis.text = element_text(size=13), axis.title = element_text(size=13), legend.text=element_text(size=13), legend.title=element_text(size=14))

## Supplementary Figure 1

| **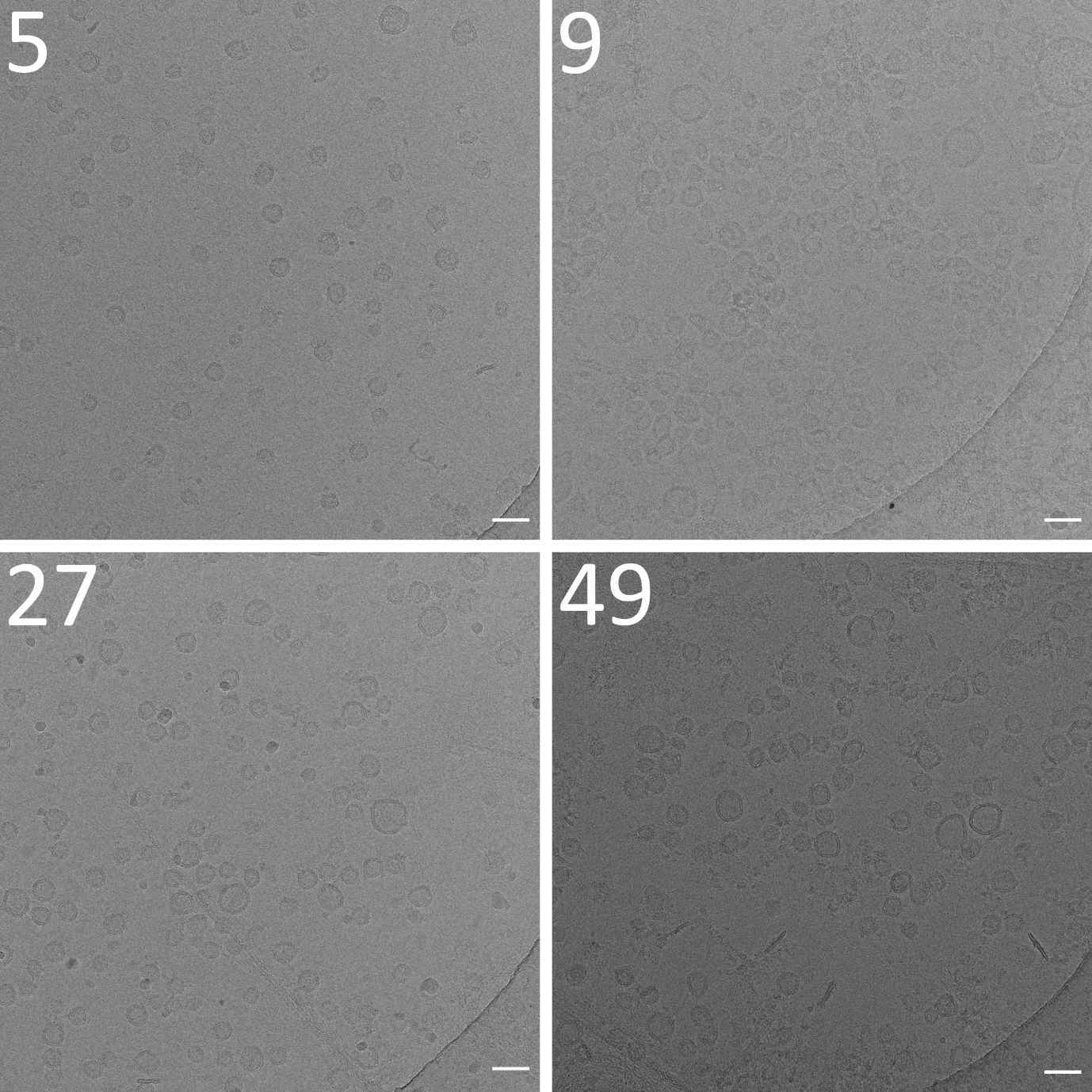** |
| --- |
| **Supplementary Figure 1. Representative Cryo-TEM images used to measure BEVs.** Number refers to the sample timepoint. Error bars are 100 nm. Additional images available as doi: 10.6084/m9.figshare.24058149 |

## Supplementary Figure 2

| **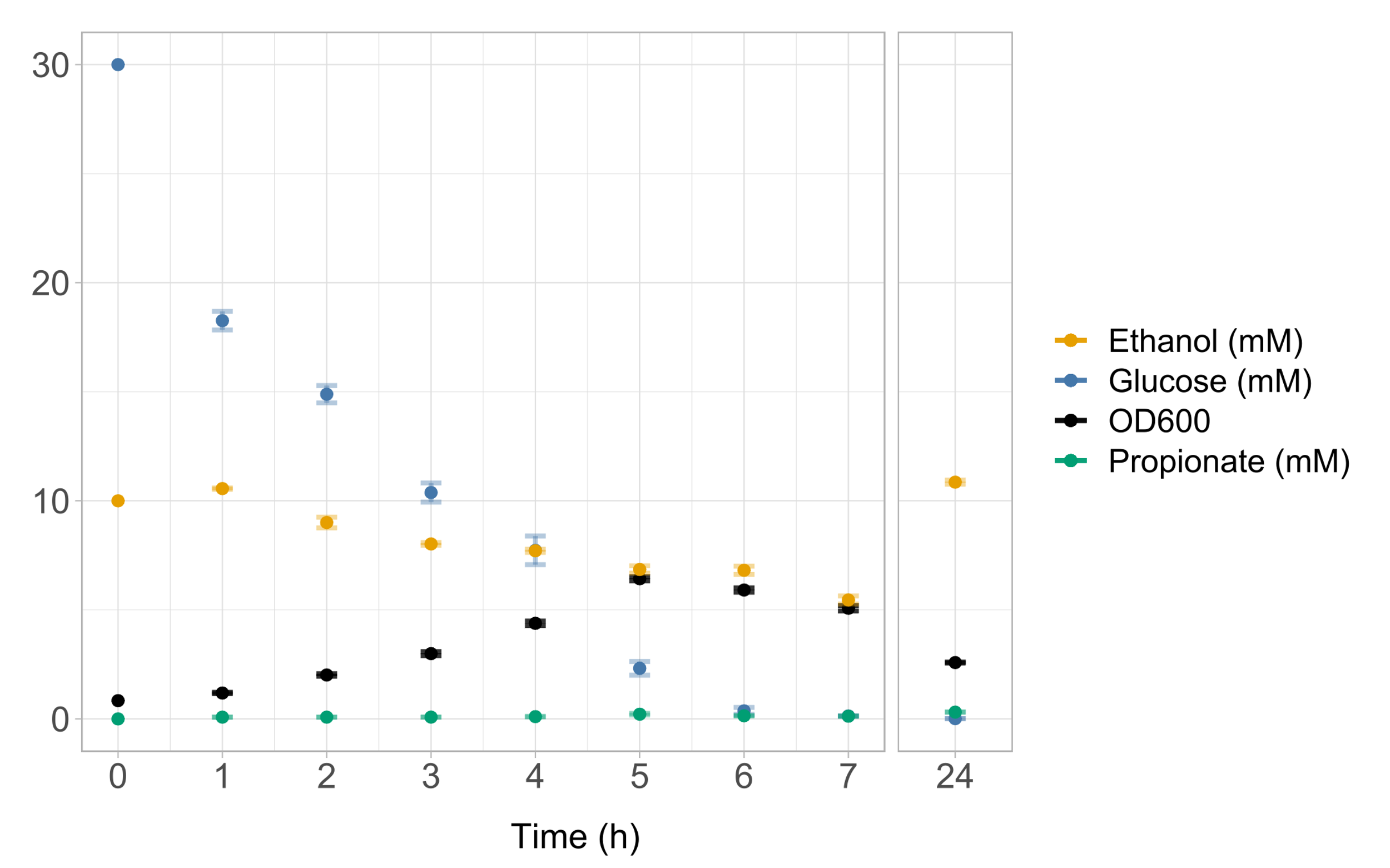** |
| --- |
| **Supplementary Figure 2. Graph showing whole range of glucose utilisation and additional analysed *B. thetaiotaomicron* metabolites.** Initial ethanol is present in the media due to presence in one of the components. Only trace levels of propionate were detected. OD600, optical density of culture at 600 nm. |

## Supplementary Figure 3

| A  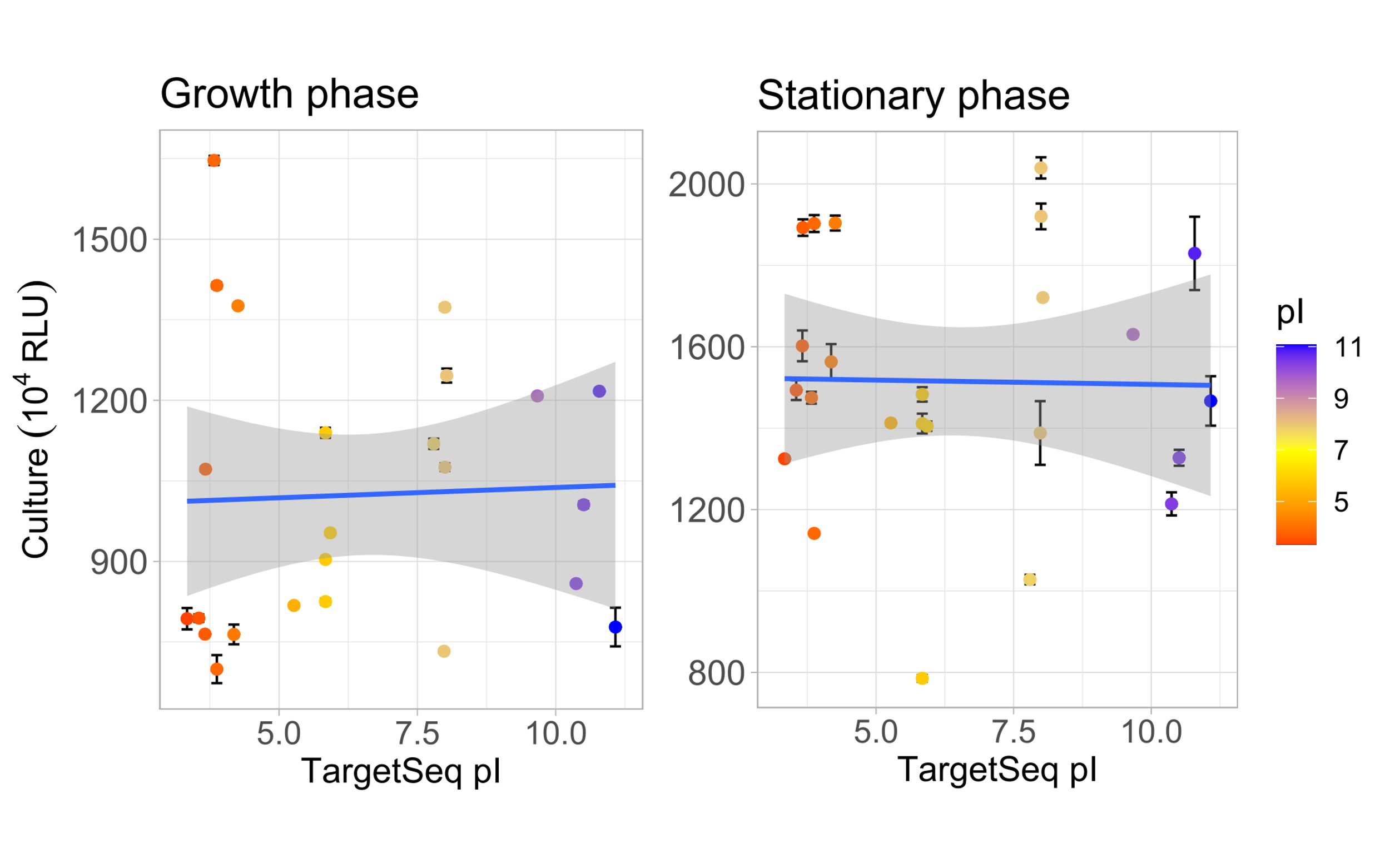 | B  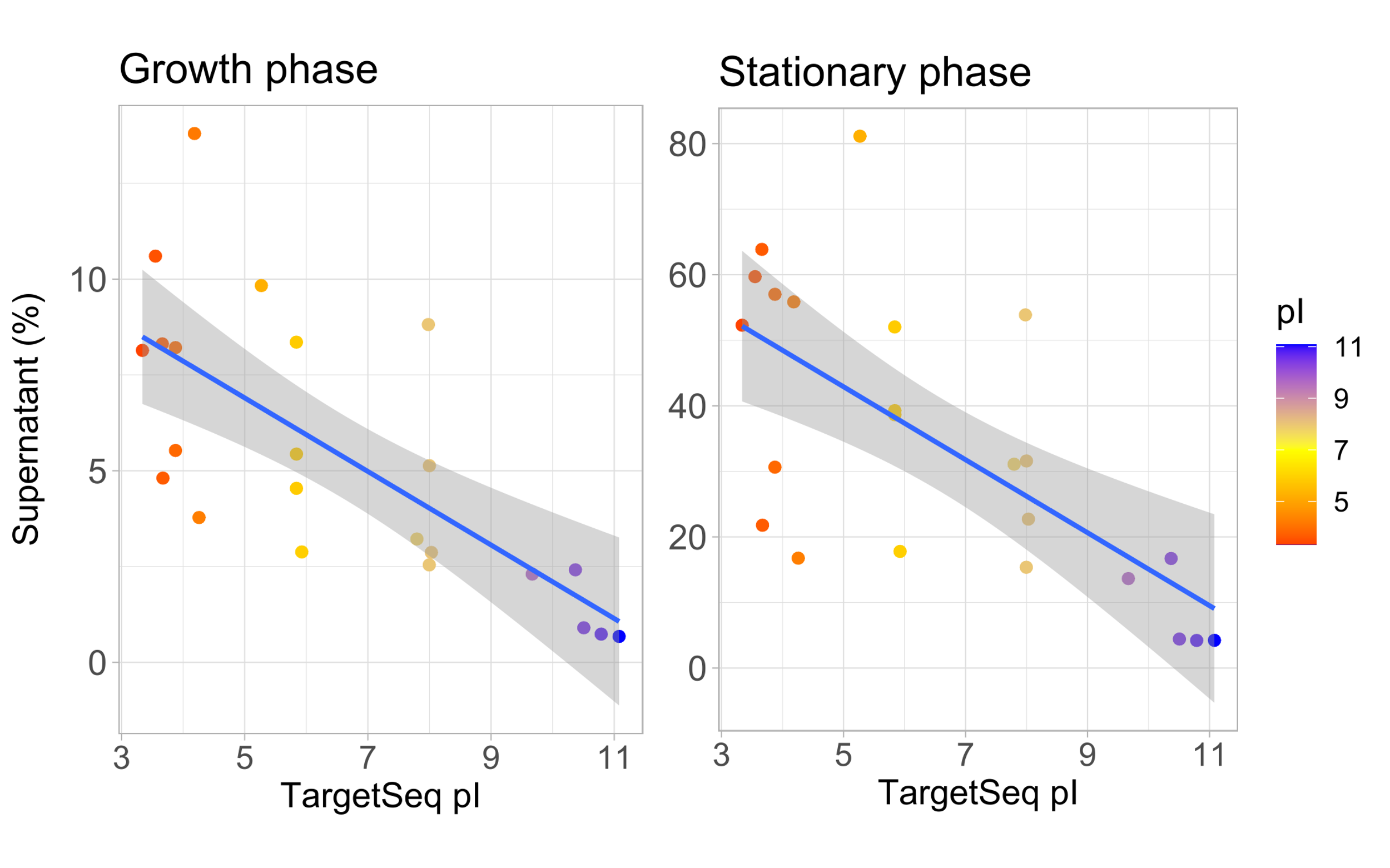 |
| --- | --- |
| **Supplementary Figure 3. Additional graphs for BEV incorporation signal peptide analysis**. Graphs show Nanoluciferase signal in whole culture (**A**) or supernatant (**B**) at two different growth phases. Supernatant graph shows percentage of exported signal. Signal is plotted against the isoelectric point (pI) of the TargetSeq. Error bars represent one standard deviation between triplicate samples. | |
